# Isozymes of AMP-Deaminase in Muscles Myasthenia Gravis Patients

**DOI:** 10.1007/s10989-016-9533-9

**Published:** 2016-05-13

**Authors:** Iwona M. Rybakowska, Stanisław Bakuła, Krystian Kaletha

**Affiliations:** 1 Department of Biochemistry and Clinical Physiology, Medical University of Gdansk, Debinki 1, 80-211 Gdańsk, Poland; 2 Department of Rehabilitation, Medical University of Gdansk, 80-211 Gdańsk, Poland

**Keywords:** AMP-deaminase, Isozyme, Myoadenylate deaminase deficiency, Myasthenia gravis, Human skeletal muscle

## Abstract

Similar symptoms observed in Myasthenia gravis (MG) can be also detected in the case of skeletal muscle AMP-deaminase deficiency. We compared the activity and expression of *AMP*-*deaminase (AMPD)* products in skeletal muscles of MG patients and MG-free individuals. The activity of AMP-deaminase in the muscles of MG patients was significantly higher than in the controls and was 2.05 µmol/min/mg protein (±0.31). The two groups differ in level of AMPD product expression. Furthermore in MG-group molecular size of isoform AMPD1 is 90 kDa in contrast to MG-free group where is present 70 kDa isoform of enzyme. The data suggests that the disturbances in transmission of neuronal signaling, taking place in the skeletal muscles of MG patients, may also change energetic metabolism of the affected muscles by changing molecular mass of isoform.

## Introduction

Myasthenia gravis (MG) is an acquired autoimmune disease of human neuromuscular system. MG manifests clinically as abnormally easy fatigability of several groups of skeletal muscles. Usually the symptoms begin in the group of extraocular muscles which results in ptosis and diplopia. Sometimes MG may be limited to these muscles but usually progresses and involves other muscular groups, including the respiratory system. The weakness and fatigability of the muscles usually grow worse in time (Porth and Matfin 2009).

MG is a consequence of antibody-mediated decrease in the number of acetylcholine receptors and resultant impairment of neuromuscular transmission. Interestingly, the majority (approximately 75 %) of MG patients present with various degree of abnormalities in the morphological structure of the persisted thymus (tumorous). This substantiates surgical removal of the thymus is one of the widely used treatment methods of MG (Porth and Matfin 2009).

AMP-deaminase (AMPD-EC3.5.4.6) is an enzyme involved in purine metabolism. It catalyses irreversible deamination of AMP to IMP, and plays an important role in the energetic metabolism of tissues, influencing the value of the adenylate energy charge in the cell (Chapman and Atkinson 1973). Moreover, AMPD participates in purine nucleotide cycle of skeletal muscles (Lowenstein and Goodman 1978). Various tissue- and stage-specific isoforms of AMPD have been identified in humans (Kaletha and Nowak 1988; Ogasawara et al. 1982). Three main AMPD isozymes, designated as M (muscle), L (liver) and E (erythrocyte) forms, are encoded by *AMPD1*, *AMPD2* and *AMPD3* genes, respectively (Morisaki et al. 1990). At least in rodents, neuromuscular junctions possess ecto-AMP deaminase activity which can dissociate extracellular ATP catabolism from adenosine formation. Ecto-AMP deaminase blunts the ATP derived adenosine A2A receptor facilitation of acetylcholine release from stimulated motor nerve endings, which may contribute to tetanic failure in myasthenic individuals (Magalhaes-Cardoso et al. 2003, Noronha-Matos et al. 2011).

Myoadenylate deaminase deficiency (mAMPDD) is a frequent, relatively benign disorder of skeletal muscles. Defect of AMPD in homo- and heterozygotic form occurs in 20 % of population. We distinguish primal and derivative form of enzymopathy. Derivative form of muscle AMP deaminase deficiency is result of skeletal muscle damage in disease muscle and nervous systems. At this people instead of reduction of AMPD activity is observed always decrease activity of creatine kinase and adenylate kinase. Whereas the primal form of defect is inherited autosomal and recessive and follows from changing in chromosome 1 (1p13–p21). It manifests as muscle fatigue following strenuous exercise (Fishbein et al. 1978, Fishbein 1985). The deficiency is an autosomal recessive disorder resulting from mutations of *AMPD1*. Its primary consequences include impairment of muscle purine metabolism and purine nucleotide cycle. Interruption of the cycle during muscle exercise decreases the adenylate energy charge of the myocyte and disturbs the rate of glycolysis and turnover of citric acid cycle (Flanagan et al. 1986; Sabina and Mahnke-Zizelman 2000; Sinkeler et al. 1987).

Results showing high level of *AMPD2* gene in hyperplastic, tumorous thymus in relation to *AMPD1* and *AMPD3* (Rybakowska et al. 2015) may substantiates AMPD participation in neurodegenerative disorder (Akizu et al. 2013). Therefore, the aim of this study was to analyse the expression level and selected physicochemical and immunological properties of AMPD isolated from skeletal muscles of patients with MG and MG-free individuals operated in one surgery clinic.

## Materials and Methods

We conduct our study on group of 25 MG-free individuals and 25 MG patients.

The data we introduce included representative samples of skeletal muscles (intercostal muscles) obtained intraoperatively from MG patients (18–38 years of age, subjected to thymectomy) and MG-free individuals (60–68 years of age, subjected to lobectomy due to lung cancer). The material was immediately washed in saline solution and frozen in liquid nitrogen.

The activity of AMPD was determined colorimetrically (Chaney and Marbach 1962) in tissue homogenates. The incubation medium, in the final volume of 0.5 ml, contained 0.1 M potassium-succinate buffer, pH 6.5, with 1 mM concentration of the substrate AMP. After equilibration of temperature at 30 °C, 25 µl of enzyme solution (containg about 5 µg of enzyme protein) was added into the incubation medium to start the reaction. The incubations were carried out for 15 min, and initial velocity of reaction was determined from the mean amount of ammonia liberated in three parallel incubations.

Protein concentration was determined according to Bradford (Bradford 1976).

Total RNA was isolated according to Chomczynski and Sacchi (1987), using 1:1 phenol–chloroform as an extracting mixture. The extracted RNA was separated on 1 % agarose gel containing 30 % formaldehyde. Expression of the AMPD genes was determined by means of RT-PCR, as described elsewhere (Roszkowska et al. 2008).

To isolate the enzyme, the tissue samples were homogenized in 3 volumes (v/w) of extraction buffer (0.089 M phosphate buffer, pH 6.5, containing 0.18 M KCl and 1 mM mercaptoethanol with addition of 1 mM phenylmethylsulfonyl fluoride (PMSF) and trypsin inhibitor), and centrifuged (20 min at 3000 g). Subsequently, SDS-PAG electrophoresis and Western blot analysis (with the use of polyclonal antibodies kindly provided by Professor R. Sabina) were performed as described elsewhere (Szydłowska et al. 2004).

The measurements of enzyme activity and mRNA levels were described as mean ± standard deviation (SD). Figures were done in Sigma Plot program. Differences between groups were analyzed by 2-sided Student unpaired *t* test.

The protocol of the study was approved by the Local Ethics Committee at the Medical University of Gdansk (decision no. NKBBN/229/2012).

## Results

**Fig. 1:**
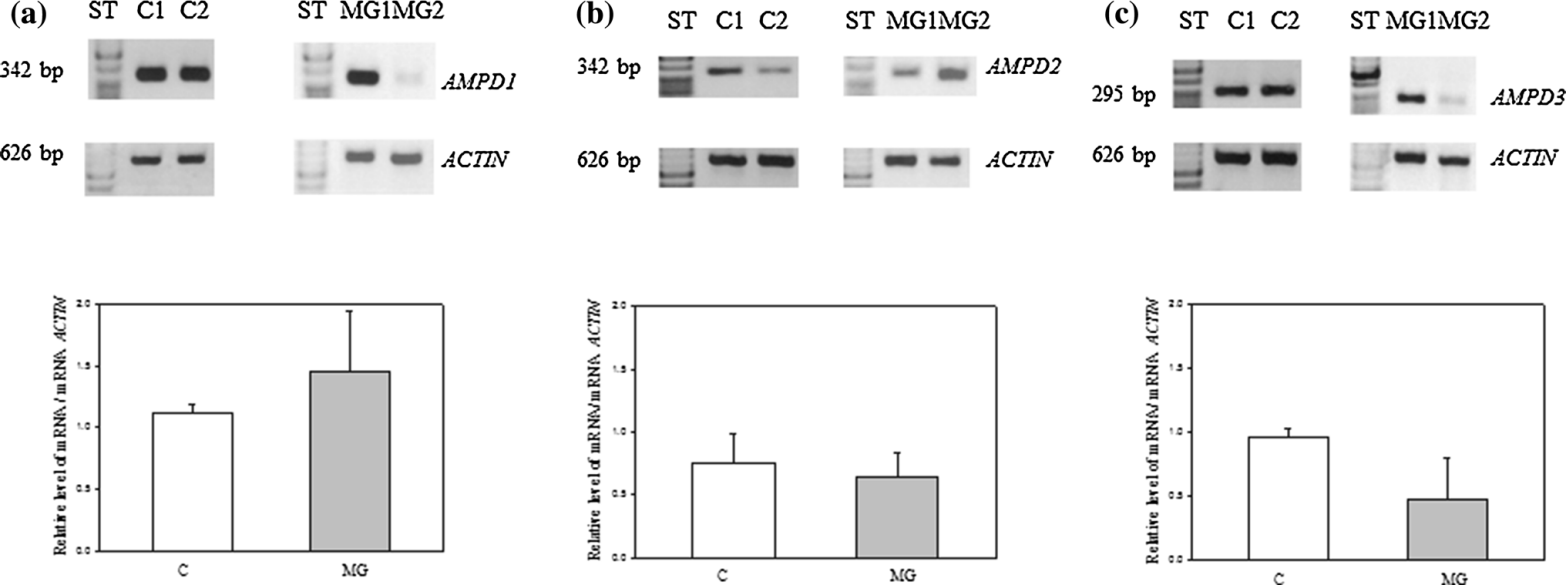
Expressions of *AMPD* family genes in representative skeletal muscles of MG patients (MG) and MG-free controls (C). mRNA of AMPD1 isoform (**a**); mRNA of AMPD2 isoform (**b**); mRNA of AMPD3 isoform (**c**). The expressions were defined as a ratio of constitutively expressed *ACTIN*. Expression levels were compared with the Student’s *t* test, n = 4 in group, (p < 0.05)

The expressions of *AMPD* family genes in skeletal muscles of MG patients and MG-free individuals are presented on Fig. 1a–c. As shown on Fig. 1a, the expression of *AMPD1* gene, physiologically most intensive in mature skeletal muscles, was even more enhanced in the material from MG patients. We did not observe similar phenomenon in the case of *AMPD3* gene. The expression of this gene in muscles of MG patients showed trend of lower expression in comparison with MG-free controls (Fig. 1c). The expression of *AMPD2* gene, extremely weak in mature skeletal muscles of healthy individuals, was also weak in the muscles of MG patients (Fig. 1b). Comparing all data showing on Fig. 1 we can say that as well as in muscles MG-free controls and MG patients the expressions of AMPD1 is highest in contrast to thymus (Rybakowska et al. 2015).

The activity of AMPD in skeletal muscle extracts of the two studied groups of patients is presented in Table 1. As shown in the Table, the activity of AMPD in the extracts obtained from MG patients (mean specific activity of about 2.05 µmol/min per mg of protein) was roughly 20 % higher than in the extracts from MG-free controls (mean specific activity about 1.64 µmol/min per mg of protein).

**Table 1:** Specific activities of AMPD in skeletal muscle extracts from MG and MG-free patients

Tissue	Specific activity (µmol/min/mg of protein)
Skeletal muscle of MG patients	2.05* (±0.31)
Skeletal muscle of MG-free controls	1.64 (±0.23)

The enzymatic activity presented as the mean value ± SD. Statistical significance verified with the Student’s *t* test, n = 4 in group, * p < 0.05

**Fig. 2:**
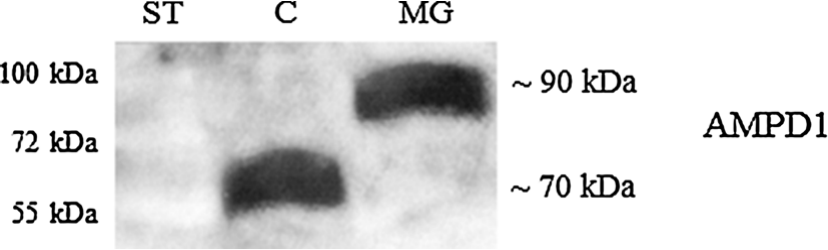
Western blot analysis of AMPD1 in skeletal muscle extracts obtained from MG patients (MG) and MG-free controls (C). Monoclonal antibodies against AMPD1 isozyme were used for immunological staining of the blot. Protein standards (ST)

Figure 2 illustrates the results of Western blot analysis performed with the prevailing enzyme isoform present in skeletal muscle extracts of the two analyzed groups of patients on representative samples (we observed the same in all studied samples). As shown on the figure, the two groups differ in immunological reaction with anti-AMPD1 antibodies. While a protein weighing about 90 kDa was labeled in the extracts from MG patients, another protein with ca. 20 kDa lower molecular mass was detected in the extracts from MG-free individuals.

## Discussion

The intracellular pool of ATP changes in response to metabolic conditions, and strenuous muscle exercise was shown to be associated with a decrease in the ATP/ADP ratio (Flanagan et al. 1986; Sinkeler et al. 1987). A reduction of cellular pH, taking place in exercising skeletal muscles, stimulates the activity of purine nucleotide cycle in order to counteract the decrease in the cellular ATP/ADP ratio (AMPD is inhibited during the initial phase of exercise due to accumulation of orthophosphate, but is reactivated by lowered pH) (Hellsten et al. 1999; Makarewicz and Stankiewicz 1974). Stimulation of purine nucleotide cycle activity augments energy production in exercising skeletal muscles [to a degree dependent on the metabolic type of the muscle (Meyer and Terjung 1979)]; the energy comes from both glycolysis and anaplerotic reaction of the citric acid cycle (Sinkeler et al. 1987; Ścisłowski et al. 1982). In view of its role in normalization of ATP to ADP ratio, prompt activation of AMPD seems vital for the maintenance of skeletal muscle contractility during periods of higher energy demand (Flanagan et al. 1986; Sinkeler et al. 1987).

Previous experimental studies showed that binding of this enzyme to myosin during intense muscle contractions changes significantly its kinetic and regulatory properties (in a substrate concentration-dependent manner (Hisatome et al. 1998; Rundell et al. 1992). Moreover, the enzyme can change its subunit composition, forming oligomers composed of products encoded by different *AMPD* genes (Fortuin et al. 1996; Mahnke-Zizelman et al. 1998). All these regulatory mechanisms are noticeable for control of the enzymatic activity during muscle contractions in vivo. The activity of AMPD is generally associated with high energy demand and sustained ATP turnover (Hancock et al. 2006).

Mammalian AMPD undergoes limited proteolysis in vitro; the degradation is limited to the N-terminal regions of the AMPD isozymes. Proteolysis of the N-terminal fragments does not reduce significantly catalytic activity of the enzyme (Mahnke-Zizelman and Sabina 2001). The use of new recombinant technologies allowed to synthesize full-size AMPD proteins with intact N-terminal fragments (Sabina et al. 1984).

Similar to the other two products of *AMPD* family genes, also AMPD1 undergoes the process of limited proteolysis. This is normally observed during purification of the enzyme and its further storage. The molecular mass of AMPD1 subunit isolated freshly from autopsied human skeletal muscle (60–72 kDa) (Mahnke-Zizelman and Sabina 2001; Stankiewicz 1981) differs markedly from that predicted on the basis of cDNA sequencing (86–87 kDa) (Sabina et al. 1984). This discrepancy is most probably a result of proteolysis taking place during the process of purification (Sabina et al. 1984). While the in vitro proteolysis of the enzyme is irreversible (Haas and Sabina 2003). It is possible that removal of fragment about 20 kDa from control sample could be ablation of N-terminal determining tissue specific properties of AMPD isoform, which may be responsible for metabolism of muscle MG patients in observed disease symptoms.

MG alters energetic metabolism of exercising skeletal muscles. Compared to the controls, patients with moderate to severe MG were characterized by significantly higher end-exercise muscle Pi/ATP ratio and significantly lower end-exercise muscle pH (Lindquist 2008; Ko et al. 2008). While the muscular weakness is mainly a consequence of impaired neuromuscular transmission, it also partially results from reduced excitation–contraction coupling (Pagala et al. 1990).

It is possible that more stable isoform AMPD1 in MG muscles may be responsible for weakness and fatigability of skeletal muscles. Further studies, because of small sample size which limit the study, are necessary to explain if the changes in myocyte metabolism, induced by impaired neuromuscular signal transmission, may also influence the physiological function of muscular AMPD isozymes.
